# Jiedu Tongluo Granules Ameliorates Post-stroke Depression Rat Model *via* Regulating NMDAR/BDNF Signaling Pathway

**DOI:** 10.3389/fphar.2021.662003

**Published:** 2021-05-20

**Authors:** Aimei Zhao, Bo Ma, Li Xu, Mingjiang Yao, Yehao Zhang, Bingjie Xue, Junguo Ren, Dennis Chang, Jianxun Liu

**Affiliations:** ^1^ Beijing Key Laboratory of Pharmacology of Chinese Materia Region, Institute of Basic Medical Sciences, Xiyuan Hospital of China Academy of Chinese Medical Sciences, Beijing, China; ^2^ Graduate School, Beijing University of Chinese Medicine, Beijing, China; ^3^ State Key Laboratory of Bioactive Substance and Function of Natural Medicines, Institute of Materia Medica, Chinese Academy of Medical Sciences and Peking Union Medical College, Beijing, China; ^4^ NICM, Western Sydney University, Penrith, NSW, Australia

**Keywords:** post-stroke depression, traditional Chinese medicine, jiedu tongluo granules, NMDAR/BDNF, neuroprotection

## Abstract

Post-stroke depression (PSD) is one of the most common stroke complications, which seriously affects stroke’s therapeutic effect and brings great pain for patients. The pathological mechanism of PSD has not been revealed. Jiedu Tongluo granules (JDTLG) is an effective traditional Chinese medicine for PSD treatment which is widely used in clinical treatment. JDTLG has a significant therapeutic effect against PSD, but the mechanism is still unclear. The PSD rat model was established by carotid artery embolization combined with chronic sleep deprivation followed by treating with JDTLG. Neurobehavioral and neurofunctional experiments were engaged in studying the neural function of rats. Histomorphology, proteomics, and western blotting researches were performed to investigate the potential molecular mechanisms related to JDTLG therapy. Oral treatment of JDTLG could significantly improve the symptoms of neurological deficit and depression symptoms of PSD rats. Proteomic analysis identified several processes that may involve the regulation of JDTLG on the PSD animal model, including energy metabolism, nervous system, and N-methyl-D-aspartate receptor (NMDAR)/brain-derived neurotrophic factor (BDNF) signal pathway. Our results showed that JDTLG could reduce glutamate (Glu) level and increase gamma-aminobutyric acid (GABA) level via regulating the NMDAR/BDNF pathway, which may play a vital role in the occurrence and development of PSD.

## Introduction

Post-stroke depression (PSD) is one of the most common psychiatric complications of stroke, racked up about 33 percent of stroke survivors (Towfighi et al., 2017). The pathogenesis of PSD is very complex, including biological and social psychological mechanisms (Wang et al., 2018). Several researches provided evidence that it may associate with the neurotransmitter system’s modulation, neuronal plasticity, neuroendocrine activation, and energy metabolism (Villa et al., 2017). However, the pathophysiological mechanisms of PSD remain far from clearness.

Glutamate, N-methyl-D-aspartate (NMDA) receptors (NMDARs), and brain-derived neurotrophic factor (BDNF) are the critical gene nodes in PSD. Glutamate is the primary excitatory neurotransmitter of the central nervous system (CNS) and plays a crucial role in maintaining the nervous system’s homeostasis and function. NMDARs are essential members of the ionic glutamate receptor family. Excess release of glutamate in the brain is one of the causes of ischemic stroke. It is known that excitotoxicity can cause neuronal death after acute stroke and is associated with overactivation of glutamate receptors (Szydlowska and Tymianski, 2010). Increased glutamate-mediated excitotoxicity could also cause PSD (Sanacora et al., 2012), as a previous study suggested that the glutamate may be involved in PSD via infarct formation (Cheng et al., 2014). Besides, BDNF plays a vital role in neuronal plasticity, cognition, learning, and memory. Numerous studies have demonstrated that the BDNF expression level in PSD patients is lower than that without depression. Moreover, antidepressants are known to improve BDNF expression in the brain, which may reduce the symptom of depression (Zhang and Liao, 2020). Inhibition of NMDARs could improve the BDNF function (Tanqueiro et al., 2018). What’s more, in central nervous system neurons, CREB phosphorylation is induced by activation of NMDARs, which lies downstream of Ca^2+^/Calmodulin dependent protein kinase activation (Deisseroth et al., 1996). Calcium-dependent nuclear signaling via CAMK4 and CREB is critical for neuroprotection (Bell et al., 2013). Thus, gaining a clearer understanding of the complex pathogenesis of PSD is essential for developing better treatments.

In recent years, Traditional Chinese Medicine (TCM), as a primary form of complementary and alternative therapy, has been recognized to be effective and safe in treating depression (Jun et al., 2014). The Chinese herbal preparation named Jiedu Tongluo granules (JDTLG) is a patented complex Chinese medicine formulation (No: 201510419571.3) (Zhao et al., 2018). It has shown that JDTLG is effective for the recovery of body function and depression in PSD patients. An earlier study showed that JDTLG could significantly improve depression-like behavior in animal stroke models (SongWT and Ren, 2015). However, the underlying mechanism was poorly understood until now. This study hypothesized that glutamate excitotoxicity is the pathogenic mechanism of PSD, and JDTLG may have an antidepressant effect and neuroprotection function in the PSD animal model. Therefore, the present research explores the therapeutic effects of JDTLG in the PSD animal model and uncovers the potential mechanism of neuroprotection through the NMDAR/BDNF signaling pathway.

## Materials and Methods

### Preparation and Analysis for JDTL Granules

JDTLG was provided by Huashen Pharmaceutical Co., Ltd. (Beijing, China, #20131230), which was composed of Panax ginseng C. A. Mey. (Ren Shen) 12.5 g/100 g, Scutellaria baicalensis Georgi (Huang Qin) 12.5 g/100 g, Ginkgo biloba L. (Yin Xing Ye) 25 g/100 g, *Hypericum perforatum* L (GuanYe Lian Qiao) 12.5 g/100 g, Gardenia jasminoides J. Ellis (Zhi Zi) 12.5 g/100 g, Gastrodia elata Blume (Tian Ma) 12.5 g/100 g, Conioselinum anthriscoides “Chuanxiong” (Chuan Xiong)12.5 g/100 g. The main compounds of JDTLG were identified according to the Chinese Pharmacopeia specifications (2010 Edition). To obtain the bioactive ingredient of ginsenoside (Ma et al., 2017), samples of JDTLG were separated on XB-C18 column (4.6 × 250 mm, 5 µm), mobile phases consisted of solvents A (acetonitrile) and B (pure water). A gradient eluting program was selected as follows: 0–35 min, 19%A with 81%B; 35–55 min, linear-gradient elution19–29%A and 81–71%B; 55–70 min, maintaining 29%A and 71%B for 15 min; 70–100 min, linear-gradient 29–38%A and 71–62%B. The flow rate was 1.0 ml/min, and the detection wavelength was 203 nm. To obtain Baicalin (Li et al., 2004), samples of JDTLG were separated on a TopsilTM C18 column (4.6 × 250 mm, 5 µm), and used methanol-water-phosphoric acid (47:53:0.2) as the mobile phase; the detection wavelength is 280 nm, the flow rate is 1.0 ml min^−1^, the column temperature controlled at 30°C. Reference substances of Ginsenoside Rg1, Ginsenoside Re, and Baicalin bought from the National Institutes for Food and Drug Control (Beijing, China). Reference substances of Ginsenoside Rb1 bought from Chengdu Pusi Biological Technology Co., Ltd. (Beijing, China).

### Animals

We used male SD (Sprague Dawley) rats (License No. SCXK 2016-0011) weighing 200–220 g supplied by the Beijing Vital River Laboratory Animal Technology Co., Ltd. Rats were reared at 25 ± 1°C and 65 ± 5% temperature and humidity, with a 12 h light-dark cycle. All rats were adapted to the environment for about one week and had free access to food and water. Try to minimize animal suffering during experiments. The Committee approved the procedures and ethics guidelines for Experimental Animal Use and Care of Xiyuan Hospital, China Academy of Chinese Medical Sciences, Beijing, China.

Rats were randomly assigned to five groups (*n* = 10): control group, model group, fluoxetine (10 mg kg^−1^) group (Liang et al., 2015; Sun et al., 2017), JDTL low group (2 g kg^−1^) and JDTL high group (4 g kg^−1^). The JDTL Granules and fluoxetine were intragastrically administrated from the first day of the surgery until the behavioral test. Rats in the control and the model group were given the same volume of drinking water. The dosage of drugs was updated according to the weight of rats weekly. Fluoxetine hydrochloride obtained from Lilly (NO. 5198A, Suzhou, China).

### Microsphere-Induced Cerebral Embolism

Microsphere-induced cerebral embolism was performed using the previously described method (Zhang et al., 2018). After intraperitoneal injection of 40 mg kg^−1^ chloral hydrate, the right common carotid artery and the rats’ external carotid were temporarily clamped with vascular clamps. The microspheres (106–212 µm in diameter, UVPMS-BY2, Cospheric, United States) were suspended in rat serum at a concentration of 1 mg ml^−1^, and 0.2 ml of this suspension was injected into the right internal carotid artery. After injection, loosened the clamp and sutured the puncture wound. The right common and external carotid arteries resumed blood supply to the brain after 2–3 s. Rats in the control group were injected with the same volume of rat serum without microspheres.

### Chronic Sleep Deprivation

The procedure of chronic sleep deprivation (CSD) was adopted from the previously published method with modifications (Alhaider et al., 2010; Wang et al., 2017; Ma et al., 2018). All rats have received a 7 days adaptation before MCE surgery and taken CSD from the third day after cerebral ischemia except the control group. The animals subjected to CSD were placed in regular containers for 16 h (16:00–8:00) per day for 4 weeks, and each CSD animal was placed on a circular platform. Six platforms were located in a rectangle container filled with room-temperature water, with 150 mm between the two platforms. During sleep deprivation, low muscle tone caused animals to fall into the water, forcing them to climb back onto the platform and stay awake. Animals in the control group were placed in identical rectangle containers without water to allow them to sleep under the same conditions. Animals were transferred to cages for the remaining 8 h/day (8:00–16:00). During the sleep deprivation, rats had free access to water and food, which hang on the container cover.

### Evaluation of Neurological Deficit

The neurological deficit scored according to Longa’s five-point scale (Longa et al., 1989). Scores were calculated for each group on days 1, 14, 28 during sleep deprivation. The following neurological deficit scoring system was used: 0, no neurological deficit (normal); 1, inability to extend forepaw fully (mild); 2, unable to move linearly and spiraling to one side (moderate); 3, unable to bear weight and fall to one side at rest (severe); and 4, no spontaneous locomotor activity or lose consciousness (critical). An uninformed researcher performed all neurological assessments.

### Behavioral Test

#### Open Field Test

The open-field test was used to assess general activity level, including locomotor activity and exploratory behavior. The test equipment was a black rectangular structure (100 × 100 × 40 cm^3)^ divided into 16 squares. Rats were initially placed in the test chamber center and observed for 5 min. Within 5 min, the total number of squares crossed with all paws was counted to assess the locomotor activity, and the number of forefeet leaving the ground was measured to evaluate exploratory behavior. The equipment was cleaned up with 10% alcohol solution after each session (Arslan et al., 2016).

#### Tail Suspension Test

The tail suspension test was carried out before and after the sleep deprivation procedure as previous reports (Kumar and Mondal, 2016). The animals were suspended 50 cm above the ground and secured with tape about 1 cm from the tail. The test lasted 6 min, and the animals’ immobility was quantified during the last 4 min of each test. Rats were considered immobile only when they were passively suspended and remained motionless.

#### Sucrose Preference Test

The sucrose preference test was performed before and after the sleep deprivation procedure as previous reports (Xu et al., 2016). First, all rats were conditioned to 1% sucrose solution, 24 h of exposure to two bottles of sucrose solution, another 24 h of exposure to one bottle of sucrose solution and one bottle of water. After the adaptation, rats were deprived of water and food for 24 h. Then sucrose preference test was conducted for 1 h. During this period, rats were housed in individual cages, with free access to two bottles, one containing 200 ml 1% sucrose solution and the other 200 ml water. The sucrose preference test was measured as a percentage of sucrose solution consumed relative to the total liquid intake.

### Hematoxylin-Eosin Staining

Histopathology was performed after the completion of behavioral tests. Rats were sacrificed after deep anesthetization with an intraperitoneal chloral hydrate injection (40 mg kg^−1^). Brain tissues were fixed in 4% paraformaldehyde at 4°C for 24 h, dehydrated in a graded series of alcohols, then embedded in paraffin, and cut into 5 µm-thick sections (Zhang et al., 2018). The sections were stained with H&E and assessed on a light microscope (Olympus FV1200, Tokyo, Japan).

### Electron Microscopy

Hippocampus slices of the ischemic hemisphere were cut into 1 mm cubes and were immediately fixed in 2.5% glutaraldehyde at room temperature for 2 h. The pieces were washed with PBS, incubated in PBS solution containing 1% osmium tetroxide for 1 h, dehydrated with ethanol, stained with 1% uranyl acetate for contrast, and embedded in EPON resin. After ultrathin sectioning by Leica EM-UC 6, the specimen sections were stained with uranyl acetate and alkaline lead citrate and observed under a transmission electron microscope (HITACHIH-7500) (Zhang et al., 2018).

### Cerebral Tissue Samples Preparation for LC-MS Analysis

The rats’ cerebral tissue was incubated in lysis buffer for 2 h at 4°C, containing NaCl 150 mmol/L, 50 mm Tris-Cl pH 7.5, 1 mm EDTA, and 1% Nonidet P-40. Then the sample was lyzed by ultrasound for 3 cycles of 30 s and centrifuged at 12,000 rpm for 15 min. The supernatant was collected and treated with 10 mM of DTT for 30 min followed by 5 mm iodacetamide for 30 min. After that, the protein sample was digested with trypsin overnight (Fornasiero et al., 2018; Wang et al., 2006).

### LC-MS/MS Analysis and Data Analysis

The peptides samples were separated on a column packed with C18 Luna beads and then analyzed using a nanoflow liquid chromatography-tandem mass spectrometry. The solvent system was made up of water (solvent A) and acetonitrile (solvent B). The peptides were eluted from 4% B to 35% B in 90 min. The mass spectrometry data was used for protein identification against the UniProt *Rattus norvegicus* protein database. Protein quantitation was analyzed using MaxQuant software. For the searches, oxidation (M) and acetylation (protein N-term) were set as the variable modifications. Two missed cleavages were allowed. Bioinformatics analysis was performed using Gene Ontology (http://www.geneontology.org/), UniProt Database (https://www.uniprot.org/), Kyoto Encyclopedia of Genes and Genomes, KEGG Resources (https://www.genome.jp/kegg/) and DAVID Bioinformatics Resources (https://david.ncifcrf.gov/conversion.jsp) (Ma et al., 2018). The mass spectrometry proteomics data have been deposited to the ProteomeXchange Consortium via the PRIDE (Perez-Riverol et al., 2019) partner repository with the dataset identifier PXD025480.

### Glu and GABA Assays

Rats were sacrificed after completion of behavioral tests, and cerebral cortex slices were homogenized and centrifuged according to the manufacturers’ instructions. Glu and GABA concentrations in the cerebral cortex were measured with Glu (EGLT-100, BioAssay systems) and GABA (201712, Bio-swamp) ELISA kits. The results are expressed as the means ± standard deviation.

### Western Blotting

The Western blotting procedures were carried out as previously described (Zhang et al., 2018). The brain tissue protein was extracted by RIPA buffer (Beyotime, China) mixed with protease and phosphatase inhibitor mixture (MCE, United States). Protein concentration was determined by using a protein assay solution (Bio-Rad). Identical quantities of protein were denatured with protein loading buffer, loaded onto 10% SDS–PAGE gels, and transferred to polyvinylidene difluoride (PVDF) membranes by electroblotting. The PVDF membranes were blocked by 5% bovine serum albumin (BSA) in TBST buffer for 1°h, and the following antibodies were used to incubate overnight at 4°C: GRIN2B (Abcam, 1:1,000 dilution), CAMK4(ABclonal, 1:1,000 dilution), CREB1 (ABclonal, 1:1,000 dilution), BDNF(ABclonal, 1:1,000 dilution), NTRK2 (Proteintech, 1:1,000 dilution) and ACTB (Sigma, 1:5,000). Reactive bands were detected using ECL detection reagent (Thermo Fisher Scientific, MA, United States) following the instructions. All the experiments reported in this study were carried out three times, and the results were repeatable.

### qPCR

According to the instructions, the total RNA in brain tissue was extracted by using Trizol reagent (Thermo Scientific, United States). A NanoDrop 2000 spectrophotometer (Thermo Scientific, United States) was used to determine the concentration and purity of RNA. The absorbance ratio (A260/280) of all samples ranged from 1.8 to 2.0, and Prime Script RT Master Mix (Takara, Dalian, China) was used to reverse transcribing 2 μg total RNA into cDNA according to the specification. qPCR was performed using the QuantiFast® SYBR® Green PCR Master Mix (Qiagen, Germany) with specific primers and expression of each sample in Light Cycler®480ⅡReal-time PCR instrument (Roche, Swiss), which was internally normalized against Actb. Primers used were as follows: Grin2b: forward 5′- AGCCCGACTAATTCCAAGGC-3′and reverse: 5′- TTG​TCT​TTC​AGG​CTC​ACG​CT-3′; Camk4: forward 5′- TGGAGGCAGTTGCTTACCTG-3′and reverse: 5′- GGT​TCC​ACA​CAC​CGT​CTT​CA -3′; Creb1: forward 5′- CCAGGGAGGAGCAATACAGC-3′and reverse: 5′- TGT​CCA​TCA​GTG​GTC​TGT​GC-3′; Bdnf: forward 5′- ATTAGCGAGTGGGTCACAGC-3′and reverse: 5′- TGG​CCT​TTT​GAT​ACC​GGG​AC-3′; Ntrk2: forward 5′- ACGGGGACCTCAACAAGTTC-3′and reverse: 5′- CTG​CGA​TTT​GCT​GAG​CGA​TG-3′; Actb: forward 5′- CCA​ACC​GTG​AAA​AGA​TGA​CC-3′ and reverse: 5′- ACC​AGA​GGC​ATA​CAG​GGA​CA-3′; Relative expression fold change was calculated using the2^−△△Ct^ method (Livak and Schmittgen, 2001).

### Statistical Analysis

All statistical data were expressed as mean ± standard deviation (SD), which were analyzed using GraphPad Prism software (San Diego, CA, United States). One-way analysis of variance (ANOVA), followed by Newman–Keuls post hoc test, was used to compare all groups’ differences. Each experiment was repeated at least three times. *p* < 0.05 was considered statistically significant.

## Results

### Qualitative Analysis of Bioactive Compounds in JDTL Granules

**FIGURE 1:**
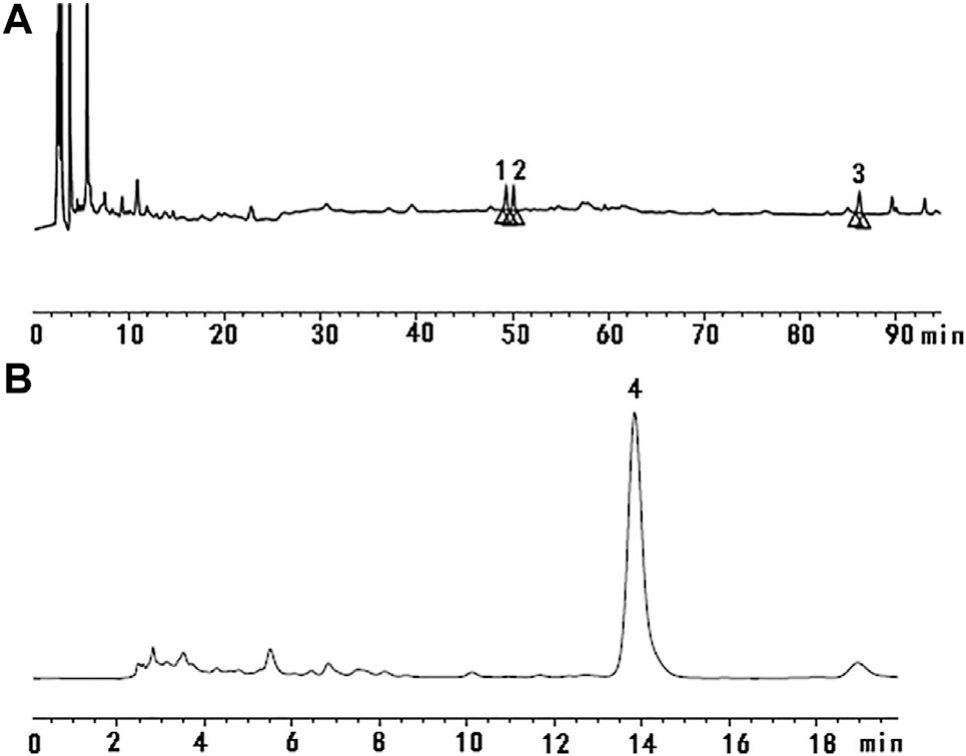
Representative chromatogram of the primary compounds in JDTLG: **(A)** 1. Ginsenoside Rg1, 2. Ginsenoside Re, 3. Ginsenoside Rb1; **(B)** 4. Baicalin.

High-performance liquid chromatography (HPLC) was used to determine the contents of representative chemical components in JDTLG. Figure 1 shows the chromatograms of the main identified components of JDTLG. In Table 1, the calibration curves’ equations and the quantitative limits of these components are determined. All calibration curves showed good linear regression (*R* ^2^ > 0.99). The results’ precision and accuracy tests are listed in Table 2. The concentration of the main compound is calculated by using the calibration curve of the internal standard.

**TABLE 1:** Linear range, *R* ^2^, and limits of quantification of calibration curve used to determine the main identified compounds.

Compounds	Linear range (μg)	Calibration curve	*R* ^2^
Ginsenoside Rg1	0.364∼3.64	Y = 2.5*10^5^X + 2,951	0.9985
Ginsenoside Re	0.317∼3.17	Y = 3.0*10^5^X − 18773	0.9999
Ginsenoside Rb1	0.324∼3.24	Y = 1.7*10^5^X − 13850	0.9995
Baicalin	0.0925∼0.925	*Y* = 4.0*10^6^ *X* − 65,619	0.9998

**TABLE 2:** Contents of the main identified compounds in JDTL Granules.

Herbs	Compounds	Contents (mg/g)
Panax ginseng C. A. Mey. (*Ren Shen*)	Ginsenoside Rg1	0.315
	Ginsenoside Re	0.343
	Ginsenoside Rb1	0.968
*Scutellaria baicalensis Georgi* (*Huang Qin*)	Baicalin	13.996

### JDTL Granules Ameliorated PSD-Induced Neurological Deficits and Depressive Symptoms

**FIGURE 2:**
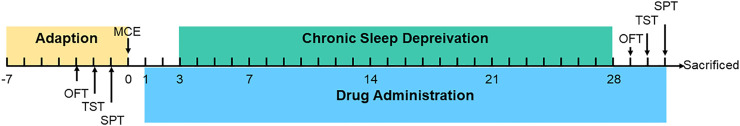
Schedule of experimental procedures.

**FIGURE 3:**
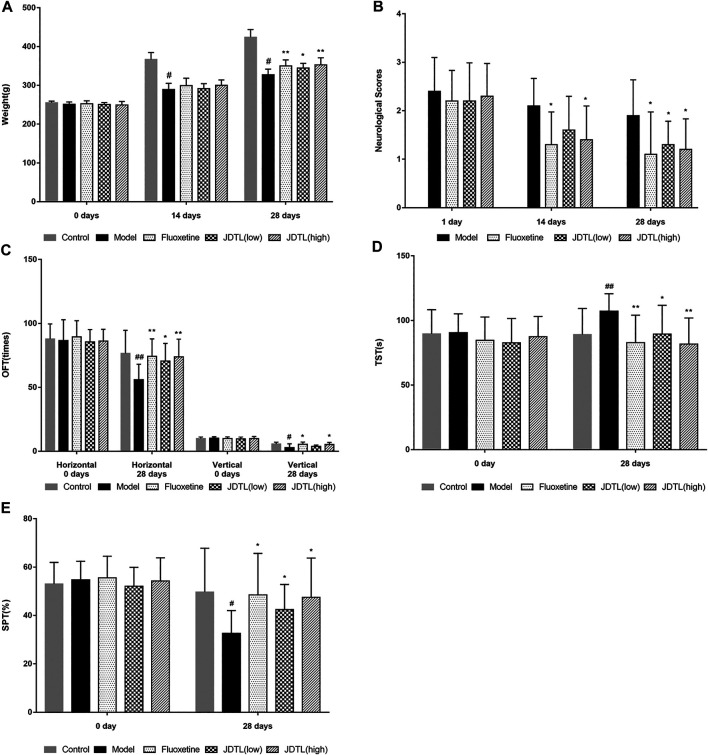
JDTL Granules Ameliorated PSD-Induced Neurological Deficits and Depressive Symptoms. **(A)** Bodyweight. **(B)** Neurological deficit scores. **(C–E)** Neurobehaviorals **(C)** OFT, **(D)** TST, **(E)** SPT. All data were expressed as mean ± SD, *n* = 10. ^##^ *p* < 0.01, ^#^ *p* < 0.05 vs. Control group, **p < 0.01, *p < 0.05 vs. Model group.

The schedule of our research procedure was shown in Figure 2. Body weights of the rats were observed every two weeks during the CSD phase. As shown in Figure 3A, MCE + CSD induced bodyweight decrease since the second week compared to the control group (*p* < 0.05). In comparison, JDTL granules and fluoxetine attenuated the fourth week’s bodyweight reduction (*p* < 0.01). Additionally, the model group's neurological deficit scores were higher on the second and the fourth week (*p* < 0.05), which exerted a delayed functional recovery. However, JDTL granules and fluoxetine treatment significantly ameliorated the neurological deficit (*p* < 0.05, Figure 3B).

What’s more, several behavioral tests were conducted to determine the antidepressant effects of JDTL granules on the PSD rats. The horizontal and vertical frequency tested the locomotor activity and exploratory behavior. Rats in the model group displayed a significant decrease in locomotor activity and exploratory behavior (*p* < 0.01 or *p* < 0.05), whereas JDTL granules and fluoxetine treatment reversed the reduction of locomotor activity (*p* < 0.05 or *p* < 0.01, Figure 3C). As for the TST, rats treated with JDTL granules and fluoxetine show a shorter immobility time compared with the model group (*p* < 0.05 or *p* < 0.01, Figure 3D). We also tested the rats’ sucrose preference and found that JDTL granules and fluoxetine could increase the rats’ sucrose preference, compared with the model group (*p* < 0.05, Figure 3E).

### Histological and Ultrastructural Changes Associated With JDTL Granules Treatment

**FIGURE 4:**
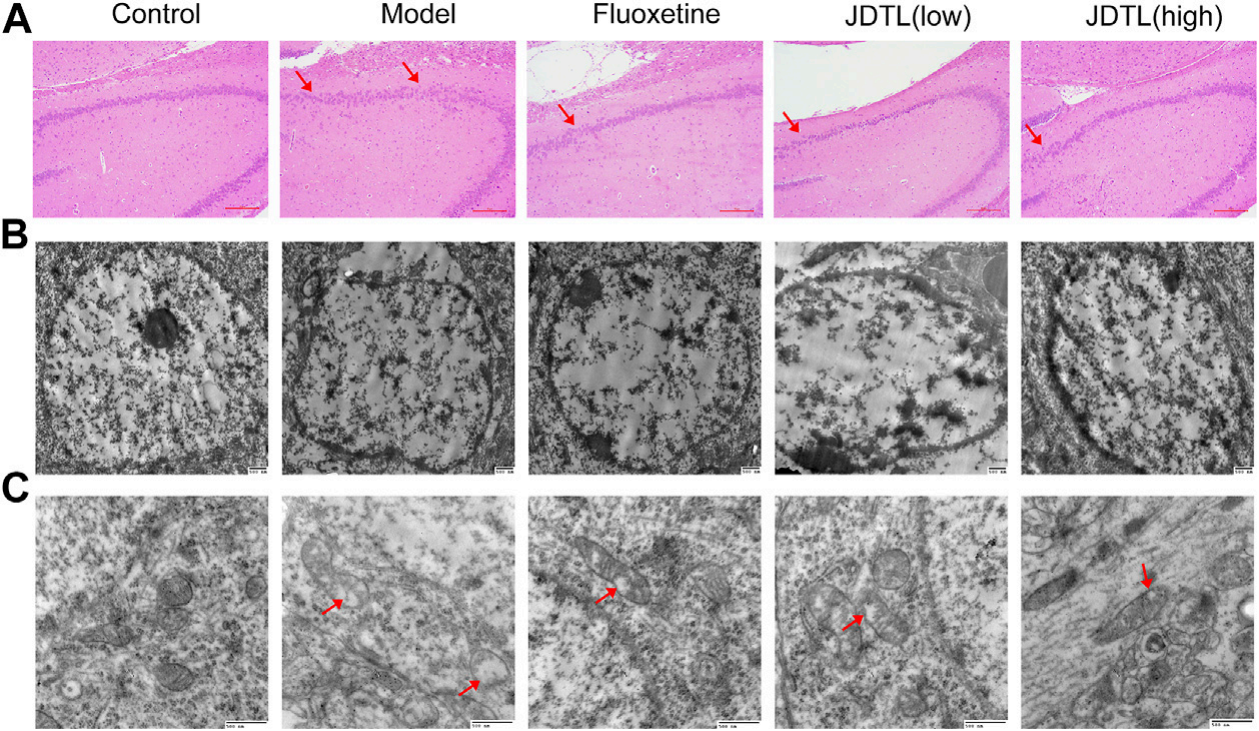
Histological and Ultrastructural Changes Associated with JDTL Granules Treatment. H&E staining of the hippocampal CA3 area [**(A)**, ×40]. Cells were structurally intact and with clear cytoplasm in the control group. In the model group, degeneration and necrosis occurred with reduced cells, scattered arrangement (arrow), and condensed and deep stained nucleus. Minor damage was observed in JDTL granules and fluoxetine groups. Ultrastructural characteristics of the nucleus [**(B)**, ×20,000] and mitochondria [**(C)**, ×40,000] in the hippocampal area. The control group neurons were with intact shape and uniform chromatin in the nucleus. Furthermore, the mitochondria were apparent and remained intact. The model group’s nucleus showed nuclear pyknosis, and the mitochondria deform, swell, and vacuolization (arrow). The treatment of JDTL granules and fluoxetine reduced the damage.

Histological changes of brain neurons can reveal the structural and functional changes of the brain. We engaged HE staining of brain neurons for all groups (Figure 4A). In the control group, the neurons in the hippocampal CA3 area were normal in morphology, structurally intact. Moreover, the cells were arranged neatly. The cytoplasm was clearly visible. However, in the model group, the cell gap was enlarged with a scattered arrangement. The number of cells was reduced. The nucleus was condensed and deep stained with degeneration and necrosis. Moreover, JDTL granules and fluoxetine treatment clearly reduced the degree of cell damage in hippocampal neurons.

Morphology study of electron microscopy in the hippocampal area generates a detailed evaluation of the nucleus (Figure 4B) and mitochondria (Figure 4C). The neurons in the control group were with intact shape and uniform chromatin distribution in the nucleus. The mitochondria were apparent and remained intact. While the nucleus of the model group showed nuclear pyknosis and nuclear membrane structure disintegrates. The mitochondria deform and swell with the sputum rupture and the vacuolization. JDTL granules treatment reduced brain tissue elements’ damage in a dose-dependent manner, and this tissue had a more viable appearance.

### LC-MS/MS Analysis

**FIGURE 5:**
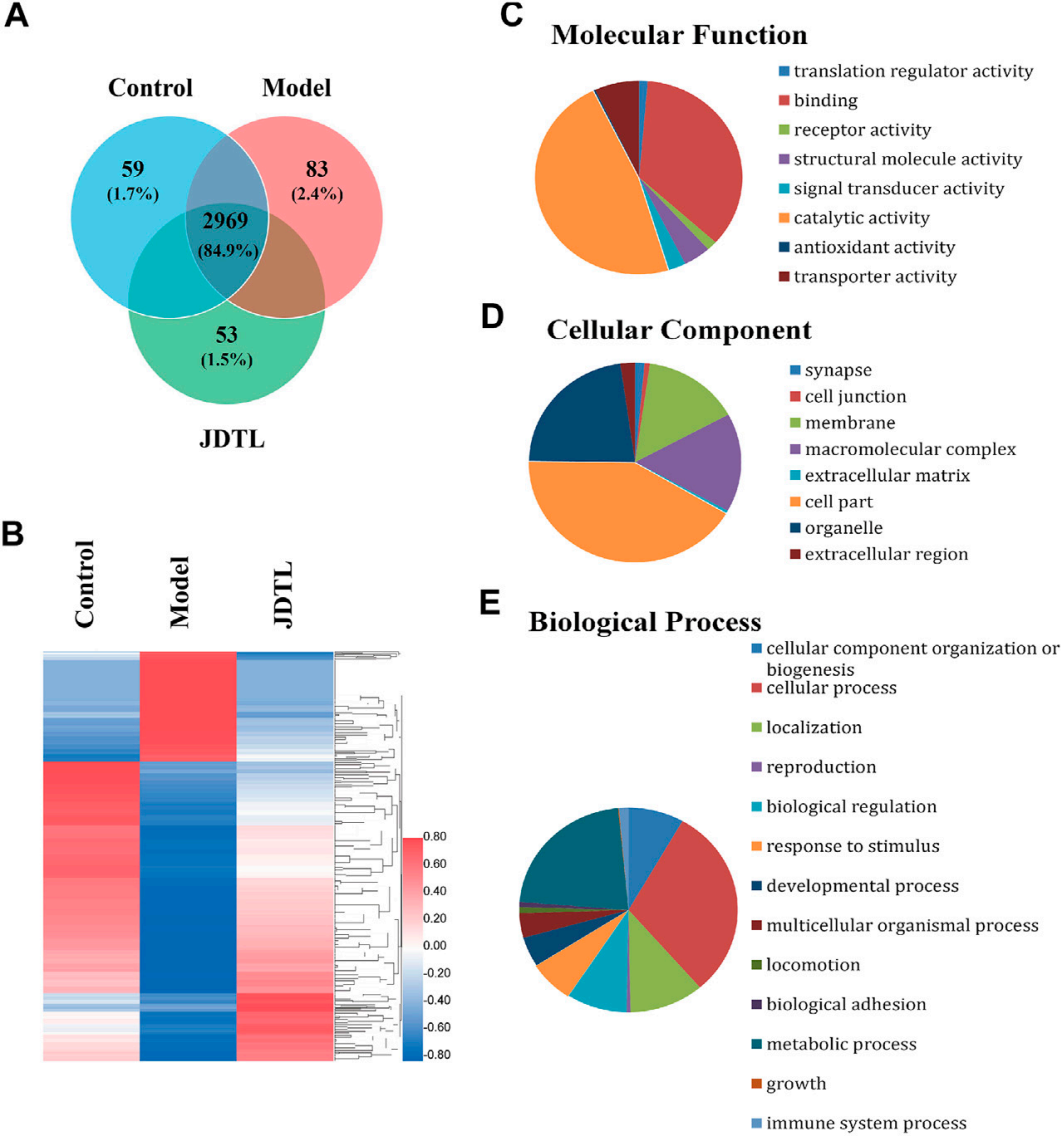
Summary statistics of LC-MS/MS analysis. **(A)** Area-proportional Venn diagram depicts the overlap of the identified proteins of Control, Model, and JDTLG groups. **(B)** Heat map analysis of the DAPs among the three groups. **(C–E)** Molecular functional assignments, cellular component and biological process of the DAPs according to gene ontology analysis.

We engaged label-free quantitative proteomic technology to study protein expression levels of PSD rat models’ brain tissue. As the statistics revealed, 3,503 non-redundant proteins were identified in the three groups of rat brain tissue. Among the proteins, 3,254, 3,231, and 3,295 proteins were detected in the control group, the model group, and the JDTL granules group. 2,969 proteins were detected in all three groups, constituting 84.9% of the total proteins (Figure 5A).

We set 2-folds as the apparent abundance alteration and detected 881 proteins that were up or down-regulated by PSD and recovered by JDTL granules (Figure 5B). These proteins were identified as the different abundance proteins (DAPs) involving in PSD. Gene ontology analysis revealed that DAPs involving in the molecular function of catalytic activity, protein binding, transporter activity, and so on (Figure 5C). The DAPs were located on several parts, including cytoplasm, membrane, organelles, cell junction, and so on (Figure 5D). Biological process analysis demonstrated that PSD might significantly affect metabolism, protein localization, biological regulation, the immune system, and other processes. Furthermore, JDTL granules may involve PSD through these processes (Figure 5E).

### Functional Enrichment of the DAPs

**FIGURE 6:**
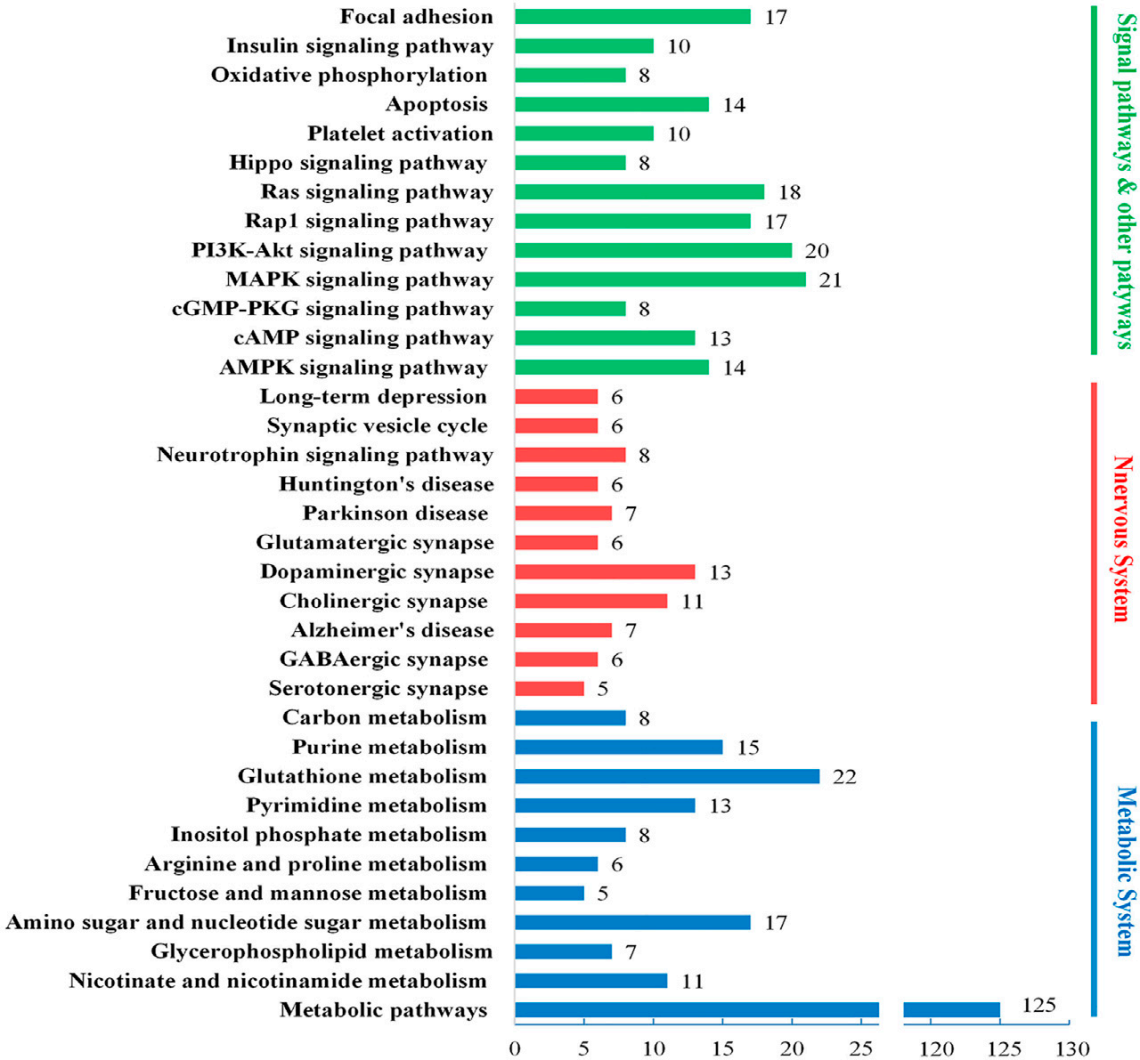
Biological process enrichment of the DAPs. Different colors represent different biological processes: blue for energy metabolism, red for the nervous system, and green for several signaling pathways. The number of every column implied the number of proteins classified in each pathway.

To further analyze the biological mechanisms involving in the DAPs, we engaged the DAVID, KEGG, and UniProt databases to study the proteins’ biological processes. As shown in Figure 6, the DAPs’ biological functions focus on three biological processes, energy metabolism, nervous system, and several signaling pathways. Based on the results of brain histochemical staining and electron microscopy analysis, we focused the therapeutic targets of JDTL granules on nerve cells’ energy metabolism and related NMDAR-CAMK4-BDNF pathways. NMDAR-CAMK4-BDNF pathways involve energy metabolism and some signal pathways, including cAMP, PI3K-Akt, Ras, and so on.

### JDTL Granules Inhibits GRIN2B and Activates BDNF Pathway in Ipsilateral Cortex

As the functional enrichment in Figure 6 shows, GABAergic synapse, Glutamatergic synapse, Cholinergic synapse, and dopaminergic synapse are detected. GABAergic synapse dysregulation has been implicated in many brain disorders. We tested the expression levels of Glu and GABA of rat brains using ELISA kits.

**FIGURE 7:**
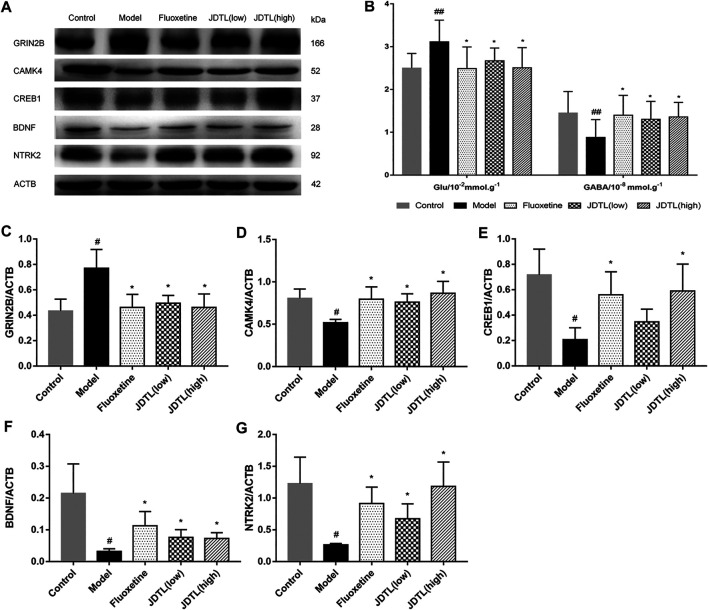
JDTL Granules Inhibits GRIN2B and Activates BDNF Pathway in Ipsilateral Cortex. **(A,C–F)** Representative immunoblots of GRIN2B, CAMK4, CREB1, BDNF, and NTRK2 in all rats’ ipsilateral cortex (*n* = 3). **(B)** The changes in Glu and GABA levels of brain tissues (*n* = 10). Data are described as mean ± SD. ^#^ *p* < 0.05, ^##^ *p* < 0.01 vs. control group. *p < 0.05 vs. model group.

The changes in Glu and GABA levels in the brain tissues were illustrated in Figure 7B (*n* = 10). It was found that the Glu levels were significantly higher in the model group (*p* < 0.01, vs. control group). JDTL granules and fluoxetine treatments notably reduced the Glu levels (*p* < 0.05, vs. model group). Additionally, the GABA was remarkably reduced in the model group (*p* < 0.01, vs. control group). JDTL granules and fluoxetine treatments significantly increased the GABA compared to the model group (*p* < 0.05).

We tested the protein expression level of the NMDAR/BDNF pathway-related proteins. And found that GRIN2B protein was significantly increased in model rats (*p* < 0.05), whereas JDTL granules and fluoxetine decreased the expression of GRIN2B (*p* < 0.05). BDNF is a crucial protein on neuron protection, and CAMK4 is a BDNF relative protein. We found that CAMK4 was a reduction in the model rats, while JDTL granules and fluoxetine increased the CAMK4 level (*p* < 0.05). What’s more, the expression of the downstream proteins of CREB1, BDNF, and NTRK2 was reduced in the model group (*p* < 0.05), whereas JDTL granules and fluoxetine treatment increased the expression level of CREB1, BDNF, and NTRK2 (*p* < 0.05) (Figures 7A,C–F ) (*n* = 3).

### Gene Expressions of NMDAR/BDNF Pathway in Ipsilateral Cortex

**FIGURE 8:**
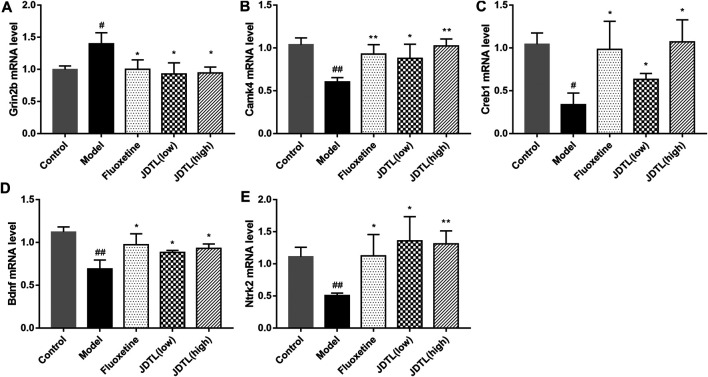
Gene Expressions of NMDAR/BDNF Pathway in Ipsilateral Cortex. **(A–E)** Relative mRNA levels of Grin2b, Camk4, Creb1, Bdnf, and Ntrk2 in the brain tissue. Data are described as mean ± SD, *n* = 3. ^##^ *p* < 0.01, ^#^ *p* < 0.05 vs Control group, **p < 0.01, *p < 0.05 vs Model group.

The relative mRNA levels of Grin2b, Camk4, Creb1, Bdnf, and Ntrk2were detected by quantitative reverse transcription-polymerase chain reaction (qRT-PCR). We observed the strong activation of the Grin2b in the model group (*p* < 0.05) and a notable reduction in both granules and fluoxetine treatment groups (*p* < 0.05, Figure 8A). On the other hand, Camk4, Creb1, Bdnf, and Ntrk2 were significantly up-regulated in granules and fluoxetine treatment groups (*p* < 0.05, Figures 8B–E) (*n* = 3), which was corresponding to the protein levels.

## Discussion

In the present study, animals were exposed to chronic sleep deprivation (CSD) after ischemic stroke to elicit experimental post-stroke depression. Sleep consists of two main stages of non-REM and REM sleep (Acosta, 2019). REM sleep is closely associated with depression. During the REM sleep phase, loss of muscle tone caused rats to fall into the water and wake up, which will cause major depressive disorders involving anxiety, anhedonia, and behavioral despair. What’s more, exposure to chronic sleep deprivation after stroke exacerbates neurological deficits, depressive-like symptoms and stimulates excitatory neurotoxicity reactions. Long-term sleep deprivation may lead to sleep disturbance and depression while reducing BDNF levels (Schmitt et al., 2016). The results indicated that the model group suffered from CSD after MCE surgery with apparent depression and neurological deficits. JDTL granules’ effect was evaluated and compared with fluoxetine (Jin et al., 2017), which showed JDTL granules had a significant effect on PSD. Our findings demonstrated that apart from the improvement of neural function recovery, JDTL granules may also notably attenuated depressive-like symptoms. However, the underlying therapeutic mechanism of JDTL granules remains unclear. Therefore, we investigated the excitatory neurotoxicity and NMDAR/BDNF signaling pathway.

We use microsphere-induced cerebral embolism combined with the chronic sleep deprivation model in this study. Embolic stroke models can cause cerebral stroke clinical symptoms, one of the models mimicking human stroke most closely (Fluri et al., 2015; Sommer, 2017). Chronic sleep deprivation can cause disruptions in circadian rhythms (Soreca, 2014), which give rise to the development of depression (Kalmbach et al., 2017; Ma et al., 2019). The combination of these two methods can reflect the disability and depression symptoms of PSD patients.

Glutamate is the primary excitatory neurotransmitter in the brain, while GABA is the primary inhibitory neurotransmitter. The balance of glutamatergic and GABAergic is essential for normal neurologic function (Guerriero et al., 2015). After a stroke, glutamate in the brain increases. Excessive glutamate stimulates glutamate receptors, leading to swelling and apoptosis of nerve cells, which in turn leads to neurological disorders (Castillo et al., 1996; Han et al., 2008). Hence, limiting secondary brain damage accompanied by excessive glutamate concentrations is an important component of stroke management (Gruenbaum et al., 2020). The glutamatergic system similarly plays a key role in mood disorders, such as anxiety (Riaza Bermudo-Soriano et al., 2012), depression (Lin et al., 2019; Sanacora et al., 2012), dementia (Butterfield and Pocernich, 2003), and other psychiatric diseases. Glutamate and GABA systems are becoming targets for the development of mood disorders drugs (Krystal et al., 2002). We observed that the change of glutamatergic synapse and GABAergic synapse were involved in the brain of post-stroke depression rats. The glutamate level was increased in the brain of MCE + CSD rats. However, the GABA level was decreased on the contrary. Correspondingly, the glutamate receptor (NMDAR) was also over-activated in the model group due to increased glutamate stimulation. Several researches indicated that antidepressants might exert their behavioral effects around the glutamate system (Sanacora et al., 2008; Chen et al., 2019; Duman et al., 2019). Following previous findings, we noticed that JDTL granules could reverse the brain’s level alteration of glutamate and GABA. Thus, the results showed that the improvement of JDTLG on PSD should be attributed to its neuroprotection via regulating excitotoxicity.

BDNF is the most abundant and widely distributed neurotrophin in the central nervous system. Animal models have been used to conduct extensive research on behavioral and emotional changes (Duman and Monteggia, 2006; Serra et al., 2017). BDNF serves as a critical transducer of antidepressants that have been linked to the antidepressant drug and the neuroplastic changes of depressive symptoms (Bjorkholm and Monteggia, 2016). The transcription of BDNF mRNA can be regulated by neuronal activity through Ca^2+^ influx, via Ca^2+^ permeable glutamate receptors (mainly NMDAR receptors) and voltage-gated Ca^2+^ channels; Zafra et al., 1991; Ghosh et al., 1994; Osteen et al., 2004). The classical cellular signaling pathway of CaM/CAMK4/CREB is closely associated with neuroprotective function. Ca^2+^ influx triggers phosphorylation of CREB, which binds to the key Ca^2+^ responsive element. The Ca^2+^ responsive element may activate BDNF transcription. The release of BDNF may stimulate NTRK2 receptors on GABAergic interneurons, which may increase GABA input to neural precursors, thus stimulating their differentiation and maturation into neurons and balancing the glutamate excitotoxicity (Waterhouse et al., 2012). Our observation suggests that GDTL Granules and fluoxetine might protect neurons via modulating the NMDAR/BDNF signaling pathway.

Although therapeutic effects of JDTL granules were observed in PSD rats, there are still some limitations. Firstly, the observation period is not long enough, and a more extended study period and sufficient samples may identify more trusted results. Secondly, fluoxetine is a representative of SSRI antidepressants. Though fluoxetine could also treat glutamate toxicity in the hippocampus (Ludka et al., 2017; Lazarevic et al., 2019), more detection of relative molecular will be better to increase the reliability of the results. Besides, we only explore the pharmacological effects of JDTLG on the glutamatergic system. The mechanism deserves further study in the future.

## Conclusion

In summary, we observed a significant neurological function recovery and antidepressants effect of JDTLG. The current investigation indicates that JDTLG can modulate excitotoxicity and alleviate depressive behavior. The beneficial effects of JDTLG treatment may be mediated by the activation of the NMDAR/BDNF signaling pathway.

## Data Availability

The datasets presented in this study can be found in online repositories. The mass spectrometry proteomics data in this article can be accessed through the following link: Project Webpage; http://www.ebi.ac.uk/pride/archive/projects/PXD025480.
